# A single amino acid change in the EGL-46 transcription factor causes defects in BAG neuron specification

**DOI:** 10.17912/micropub.biology.000224

**Published:** 2020-02-25

**Authors:** Rasoul Godini, Kasper Langebeck-Jensen, Roger Pocock

**Affiliations:** 1 Development and Stem Cells Program, Monash Biomedicine Discovery Institute and Department of Anatomy and Developmental Biology, Monash University, Melbourne, Victoria 3800, Australia; 2 Biotech Research and Innovation Centre, University of Copenhagen, Ole Maaløes Vej 5, Copenhagen, Denmark

**Figure 1:**
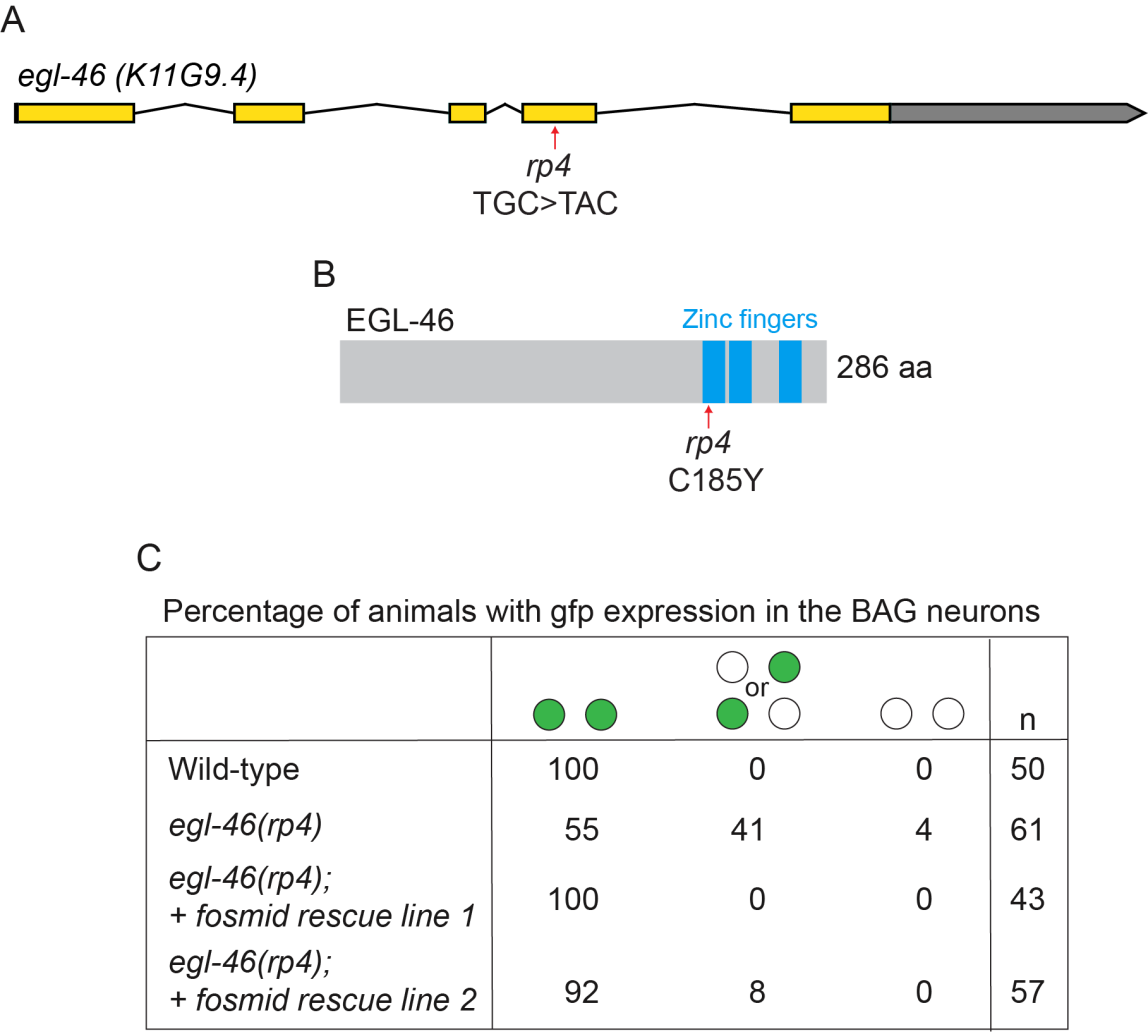
(A) Schematic of the *egl-46* genomic locus showing the *rp4* genetic lesion (TGC>TAC). (B) Schematic of the EGL-46 protein showing the amino acid change (C185Y) caused by *rp4.* (C) Quantification of *Pgcy-33::gfp* expression defects in *egl-46(rp4)* animals. Transgenic expression of a fosmid (WRM0636bB06) containing the entire *egl-46* genomic locus rescues the loss of *Pgcy-33::gfp* expression in the BAG neurons observed in *egl-46(rp4)* mutant animals. Circles indicate *gfp* expression level in the pair of left and right BAG neurons.

## Description

The BAG neurons control multiple aspects of *Caenorhabditis elegans* behavior, such as sensing environmental gases (oxygen and carbon dioxide), regulation of systemic fat levels and egg laying (Brandt *et al.* 2012; Guillermin *et al.* 2011; Juozaityte *et al.* 2017; Zimmer *et al.* 2009). To identify factors that control BAG specification, we performed a forward genetic mutagenesis screen using the *Pgcy-33::gfp* reporter, which is exclusively expressed in the BAG neurons. We isolated a new allele (*rp4)* that exhibits a loss of *Pgcy-33::gfp* expression. Using the one-step whole-genome sequencing and SNP mapping strategy (Doitsidou *et al.* 2010) we mapped the genetic lesion to the *egl-46* gene, encoding a zinc finger transcription factor homologous to mammalian INSM1/2, which we had previously shown to be important for BAG specification (Rojo Romanos *et al.* 2015). The new lesion we identified *egl-46(rp4)* (TGC>TAC) causes a single amino acid change in a highly conserved cysteine residue (C185Y) that lies in the first zinc finger domain of EGL-46, which would be predicted to affect DNA binding. Analysis of *Pgcy-33::gfp* expression in the *rp4* allele reveals that it exhibits the same phenotype as the previously published *rp13* deletion allele, which is an out-of-frame deletion that removes the zinc finger domains (Rojo Romanos *et al.* 2015). Therefore, *rp4* acts as a strong loss-of-function/null allele and may be of use to those researchers interested in elucidating additional functions of EGL-46.

## Methods

In the forward genetic screen, the BAG reporter strain *Pgcy-33::gfp; Pdop-3::rfp* was mutagenized using ethyl methanesulfonate. Mutants with decreased GFP expression in the BAG neurons were isolated using the automated COPAS biosorter platform. The one-step whole-genome sequencing and SNP mapping strategy (Doitsidou *et al.* 2010) was used to identify the genetic lesion of the isolated *rp4* allele. Phenotypic analysis of *Pgcy-33::gfp* BAG expression was performed as described previously (Rojo Romanos *et al.* 2015).

## Reagents

RJP22 *rpIs3(Pgcy-33::gfp); vsIs33(Pdop-3::rfp)*

RJP56 *egl-46(rp4); rpIs3(Pgcy-33::gfp); vsIs33(Pdop-3::rfp)*

RJP4585 *egl-46(rp4); rpIs3(Pgcy-33::gfp); vsIs33(Pdop-3::rfp); rpEx2046 (WRM0636bB06) 1ng/µl + Punc-122::gfp 30ng/µl* Line 1

RJP4586 *egl-46(rp4); rpIs3(Pgcy-33::gfp); vsIs33(Pdop-3::rfp); rpEx2047 (WRM0636bB06) 1ng/µl + Punc-122::gfp 30ng/µl* Line 2

Strains will be available at the CGC.
